# Impact of Nutritional and Diabetological Education on Glycemic Control and Obstetric and Perinatal Outcomes in Gestational Diabetes Mellitus

**DOI:** 10.3390/nu18030513

**Published:** 2026-02-02

**Authors:** Alba Yuste Gómez, Mª del Pilar Ramos Álvarez, Beatriz Barquiel, José Luis Bartha

**Affiliations:** 1 Department of Maternal Fetal Medicine, Hospital Universitario La Paz, 28046 Madrid, Spain; 2 Faculty of Health Sciences, Catholic University of Ávila (UCAV), Calle Canteros s/n, 05005 Ávila, Spain; 3 Department of Chemistry and Biochemistry, Faculty of Pharmacy, CEU San Pablo University, 28925 Madrid, Spain; pramos@ceu.es; 4 Diabetes Unit, Department of Endocrinology and Nutrition, Hospital Universitario La Paz, 28046 Madrid, Spain; beatriz.barquiel@gmail.com

**Keywords:** gestational diabetes mellitus, nutritional education, metabolic control, lifestyle, pregnancy, maternal-fetal outcomes

## Abstract

**Background/Objectives**: Gestational diabetes mellitus (GDM) is one of the most common metabolic complications during pregnancy. Nutritional and diabetological education constitutes the cornerstone of treatment; however, its actual impact according to maternal knowledge levels requires further evaluation. The objectives of this study were to assess the influence of maternal dietary and lifestyle knowledge on the metabolic control of women diagnosed with GDM and to analyze the effectiveness of nutritional and diabetological education received during pregnancy in achieving favorable obstetric and perinatal outcomes. **Methods**: A prospective observational cohort study was conducted at a tertiary referral center in women diagnosed with GDM. Participants completed a specific questionnaire to evaluate dietary and lifestyle knowledge relevant to glycemic control. Pregnancy follow-up included anthropometric measurements, maternal biochemical parameters—including oral glucose tolerance tests—and maternal-fetal obstetric outcomes, analyzed in relation to knowledge levels and education received. **Results:** Results showed that women with lower nutritional knowledge exhibited higher body weight, body mass index, and glucose levels in GDM diagnostic tests. Higher knowledge levels were associated with improved metabolic control. Nutritional and diabetological education during pregnancy proved beneficial, with better maternal-fetal outcomes observed, particularly among women who received reinforced education. **Conclusions**: Dietary and lifestyle knowledge significantly influenced GDM metabolic control. Nutritional education before and during pregnancy is key to optimizing glycemic management and improving maternal-fetal outcomes, supporting the need for preventive and educational programs targeting women with risk factors.

## 1. Introduction

Gestational diabetes mellitus (GDM) is defined as any degree of hyperglycemia first diagnosed during pregnancy that does not meet the criteria for preexisting overt diabetes [1,2]. It is one of the most common metabolic complications of pregnancy, with a rising global prevalence influenced by factors such as maternal age, body mass index, family history of diabetes, and the diagnostic criteria applied [1,3].

GDM is associated with an increased risk of maternal complications, including hypertensive disorders of pregnancy, higher cesarean section rates, and an elevated likelihood of developing type 2 diabetes mellitus postpartum [1,4]. From a perinatal perspective, maternal hyperglycemia is linked to macrosomia, neonatal hypoglycemia, preterm birth, and long-term metabolic risk in the offspring, partially mediated by fetal programming mechanisms [5,6].

Current clinical guidelines recommend lifestyle modification as the first-line treatment following a GDM diagnosis, with nutritional and diabetological education, together with the promotion of physical activity, forming the cornerstone of therapeutic management [2,7,8,9]. The primary goal is to achieve adequate metabolic control to reduce the incidence of obstetric and perinatal complications. However, treatment response is not uniform among women diagnosed with GDM, suggesting that individual factors, such as dietary and lifestyle knowledge, may influence adherence to recommendations and metabolic outcomes.

Several studies have shown that a balanced diet, characterized by control of the glycemic index and total carbohydrate intake, appropriate meal distribution, and adequate macronutrient quality, contributes to improved glycemic control in women with GDM [10,11,12]. In addition, regular moderate-intensity physical activity has been associated with reductions in fasting and postprandial glucose levels, as well as a lower need for pharmacological treatment [13].

Nevertheless, the effectiveness of these recommendations largely depends on the understanding and practical application of dietary and lifestyle principles by pregnant women. Insufficient or incorrect knowledge regarding nutrition, carbohydrate management, fat intake, or exercise can hinder optimal metabolic control, even among women receiving health education during pregnancy [14,15].

Although nutritional and diabetological education is part of standard GDM management, there is limited evidence systematically evaluating its effectiveness according to knowledge levels and its impact on metabolic control and obstetric and perinatal outcomes. A previous study conducted by our group demonstrated that pregestational education plays a fundamental role in achieving optimal maternal and fetal outcomes [16]. Assessing this relationship helps identify areas for improvement in educational programs and reinforces the need for structured interventions before and during pregnancy, especially in women with preexisting risk factors.

Therefore, the present study aimed to evaluate the influence of maternal dietary and lifestyle knowledge on metabolic control in women diagnosed with GDM, as well as to analyze the effectiveness of nutritional and diabetological education received during pregnancy in achieving favorable obstetric and perinatal outcomes.

## 2. Materials and Methods

This study was conducted at a tertiary referral center and was approved by the local ethical committee.

### 2.1. Study Population

The cohort comprised 51 pregnant women diagnosed with gestational diabetes mellitus (GDM), who, after providing written informed consent, completed a questionnaire specifically designed to assess knowledge of nutrition related to glycemic control and lifestyle (see Questionnaire in Supplementary Materials). The questionnaire was administered between 24 and 28 weeks of gestation.

All participants received clinical follow-up throughout pregnancy, including biochemical assessments, as well as documentation of obstetric and perinatal outcomes. Biochemical and perinatal results were compared according to the participants’ nutritional knowledge level (correct versus incorrect questionnaire responses). Additionally, differences in these outcomes were analyzed between women who required insulin treatment and those who achieved glycemic control through diet alone.

Inclusion criteria: (1) maternal age over 16 years; (2) provision of written informed consent; (3) comprehension of the language; (4) gestational age between 24 and 28 weeks; and (5) diagnosis of GDM. Exclusion criteria: (1) refusal to provide informed consent; (2) repeated vomiting after oral glucose tolerance testing, preventing completion of diagnostic tests; (3) known fetal anomaly; and (4) pregestational diabetes.

### 2.2. Diagnosis of Gestational Diabetes

GDM was diagnosed according to a two-step strategy and the recommendations of the Spanish Group for Diabetes and Pregnancy (GEDE) [17].

### 2.3. Patient Recruitment

Pregnant women diagnosed with GDM were recruited from the obstetrics and diabetological education clinics at La Paz University Hospital, Madrid. The 51 enrolled women received pregnancy follow-up, and data were collected on their nutritional knowledge related to glycemic control and lifestyle.

### 2.4. Questionnaire

A specific questionnaire was developed to assess the knowledge of pregnant women with GDM regarding glycemic control and the nutrition involved, as well as their lifestyle (see Questionnaire in Supplementary Materials).

The questionnaire items were organized into sections, as detailed in Table 1. In the fifth section, some questions were designed to be answered exclusively by women requiring insulin treatment.

### 2.5. Data Collection and Evaluation of Maternal and Neonatal Parameters

Data on nutritional knowledge related to glycemic control, as well as demographic information, family history, and medical history, were collected through completion of the questionnaire between 24 and 28 weeks of gestation. Anthropometric information was obtained during obstetric and gynecology follow-up visits attended by the participants.

Biochemical data and perinatal and neonatal outcomes were retrieved from the electronic medical records. Venous blood samples, collected during pregnancy follow-up visits and the oral glucose tolerance test (OGTT), were processed for biochemical analysis following established procedures at the center.

Maternal parameters evaluated: Clinical parameters were assessed during the second trimester and included the results of GDM screening and diagnostic tests: fasting plasma glucose, O’Sullivan test at 1 h, and 100 g oral glucose load with measurements at 1, 2, and 3 h. Basal insulin, HbA1c, ferritin, total proteins, hemoglobin, total cholesterol, HDL and LDL cholesterol, vitamin D, current weight, pre-pregnancy weight, and body mass index (BMI) were also analyzed.

Maternal pregnancy outcomes: Blood pressure, final weight, total weight gain, gestational age at delivery, mode of delivery, supplement intake, reason for hospital admission, and discharge diagnosis were recorded. Postpartum assessment included a 75 g OGTT with measurements at 30 min, 1 h, and 2 h, as well as postpartum HbA1c and basal insulin.

Neonatal outcomes: Neonatal weight, length, head circumference, weight percentile, and Apgar scores at 1 and 5 min were collected.

### 2.6. Statistical Analysis

Statistical analysis was performed using IBM SPSS Statistics software, version 30.0 and graphical representations were generated with GraphPad Prism 9. A descriptive analysis was conducted for all study variables, with data presented as histograms or pie charts depending on whether the variable was quantitative or categorical.

Normality tests for quantitative variables were performed using the Kolmogorov–Smirnov or Shapiro–Wilk tests to determine whether parametric or non-parametric tests were appropriate for hypothesis testing. The significance level was set at 95% (*p* < 0.05).

Comparisons of means were conducted between women who answered the questionnaire items correctly or incorrectly and who required dietary or insulin treatment, using the Mann–Whitney U test or Kruskal–Wallis test depending on whether the categorical variable had two or more groups. Correlations between variables were assessed using Spearman’s correlation analysis, as most variables did not follow a normal distribution.

In order to evaluate the potential influence of confounding factors, a multivariate analysis using binary logistic regression was performed. Insulin requirement was used as the dependent variable, and demographic variables including educational level, occupation, parity, and body mass index (BMI) were included as independent variables.

Sample size was calculated considering basal insulin as the primary outcome variable, total daily glucose measurements as the most representative question, and the results of a previous pilot study. Using an alpha level of 0.05 and a power (1 − β) of 0.80, the required sample size was 12 women in the incorrect answer group and 24 in the correct answer group, resulting in a total of 36 women with gestational diabetes. A higher number of participants was included to account for potential withdrawals.

### 2.7. Educational Sessions

Pregnant women who required pharmacological treatment with insulin due to insufficient glycemic control through dietary measures received a two-step diabetological education: initially in the obstetrics and gynecology clinic, and subsequently in the diabetological education unit.

## 3. Results

Within the study cohort of 51 pregnant women diagnosed with GDM, 7 required insulin treatment (7/51; 13.7%). Of this cohort, 38 women delivered at the hospital, from whom perinatal data were obtained.

### 3.1. Medical and Obstetric History

Among the women with GDM who participated in the study, 17.6% (9/51) had a chronic disease. A total of 27.5% (14/51) reported having some type of allergy. Regarding the presence of family history of diabetes, 68% (34/51) reported having affected relatives, with 55.9% (19/34) being first-degree relatives.

Regarding previous pregnancies with glucose abnormalities, 35.3% (18/51) of the women had experienced such complications. Of these, 61.1% (11/18) were able to manage glucose levels with diet and exercise, while 38.9% (7/18) required insulin treatment. Among the seven patients who required insulin in a prior pregnancy, 42.9% (3/7) received NPH insulin, another 42.9% (3/7) received rapid-acting insulin, and 14.3% (1/7) received a combined regimen of rapid- and long-acting insulin.

Of the 18 women with previous glucose issues, 61.1% (11/18) performed pre- and postprandial glucose monitoring, whereas 38.9% (7/18) did not adhere to these recommendations.

Regarding the mode of delivery in previous pregnancies, 38.2% (20/51) reported a vaginal birth, and 17.6% (9/51) underwent cesarean section. In the current pregnancy, 17.6% (9/51) monitored pre- and postprandial glucose, 47.1% (24/51) monitored pre- and postprandial glucose when symptoms were present, 9.8% (5/51) measured glucose before main meals, 7.8% (4/51) did not monitor glucose at all, and 17.6% (9/51) did not respond to this question.

The mean number of children among the participants was 1.84 ± 0.96 (range: 1–5), and the mean birth weight of the previous child was 3314 ± 652.12 g (range: 1500–4700 g).

### 3.2. Pre-Pregnancy Maternal Anthropometry, Maternal Biochemical Parameters, and Maternal and Neonatal Outcomes

Maternal biochemical parameters evaluated during the second trimester of pregnancy (24–28 weeks) in the full cohort of women with GDM are shown in Table A1 of Appendix A. The same table also presents pre-pregnancy maternal anthropometry and perinatal, maternal, and neonatal outcomes.

### 3.3. Relationship Between Pre-Pregnancy Maternal Weight and Gestational Maternal and Neonatal Outcomes

**Figure 1:**
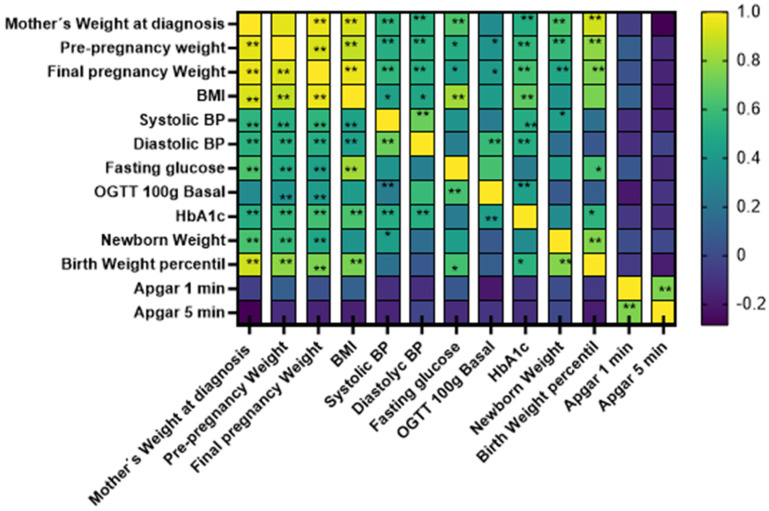
Heat map of the associations between anthropometric, biochemical, and perinatal variables. The graph shows correlations using Spearman’s rho. Correlations with a significant *p*-value are indicated with an asterisk. * *p* < 0.05; ** *p* < 0.01.

A correlation analysis was conducted between anthropometric and biochemical data and maternal and fetal outcomes with glucose levels and glucose tolerance. To simplify the presentation of these results, a heat map was generated including especially parameters showing significant correlations, as shown in Figure 1.

As observed, the results indicate a positive association between pre-pregnancy, mid-pregnancy, and end-of-pregnancy maternal weight with blood pressure, fasting glucose, fasting glucose during the 100 g oral glucose tolerance test, HbA1c, neonatal weight, length, and weight percentile, as well as correlations among all these parameters (Figure 1).

### 3.4. Relationship Between Knowledge of Nutrition Involved in Glycemic Control and Lifestyle with Maternal and Perinatal Parameters

This analysis was performed by comparing each questionnaire item with the evaluated parameters to determine whether they were associated with the knowledge and lifestyle of the participants. The questionnaire included questions on: (A) nutritional knowledge related to glycemic control, (B) insulin treatment, and (C) physical activity.

The evaluated parameters were divided into **initial parameters**: (1) maternal weight at the time of pregnancy, (2) pre-pregnancy maternal weight, (3) body mass index (BMI), (4) fasting glucose, (5) O’Sullivan test glucose, (6) fasting glucose in the oral glucose tolerance test (OGTT), (7) 1 h OGTT glucose (100 g), (8) 2 h OGTT glucose (100 g), (9) 3 h OGTT glucose (100 g), (10) area under the glucose curve from the OGTT, (11) ferritin, (12) total daily glucose measurements (number), (13) total proteins, (14) basal insulin, (15) total cholesterol, (16) HDL cholesterol, (17) LDL cholesterol, and (18) triglycerides; and **final parameters**: (1) final maternal weight, (2) systolic blood pressure, (3) diastolic blood pressure, (4) gestational age at delivery, (5) neonatal weight, (6) neonatal length, (7) Apgar score at 1 min, (8) Apgar score at 5 min, and (9) neonatal weight percentile.

Initially, the study divided women into those who answered questionnaire items correctly (C) or incorrectly (I) regarding nutritional knowledge related to glycemic control. This approach allowed the identification of differences in initial parameters between women with higher versus lower knowledge on the role of nutrition in glycemic control (Table 2) and whether these differences could affect the final evaluated parameters according to the type of treatment required. The results are presented in Table 3.

### 3.5. Association Between Women’s Knowledge (Correct/Incorrect Responses) and Initial and Final Evaluated Parameters

#### 3.5.1. Questions on Nutritional Knowledge Related to Glycemic Control

The following section shows the differences in mean values of parameters that demonstrated statistically significant differences between women who answered the questionnaire items correctly or incorrectly.

First, differences in initial parameters are presented according to the specific questions asked (Table 2). Questions can be referenced by their number in the questionnaire; examples include:

- Question 20: How many glucose measurements do you perform per day?
- Question 22: What do we mean by hypoglycemia?
- Question 23: Which glucose level is considered hypoglycemia?
- Question 24: Which of the following are symptoms of hypoglycemia?
- Question 25: What is the glycemic index of a food?
- Question 27: How many grams of carbohydrates are considered one serving?
- Question 28: Which of the following foods has a higher glycemic index?
- Question 29: Which of the following foods has a higher glycemic load?
- Question 31: In a hypoglycemic situation, which foods would you consume first?
- Question 32: What is a simple carbohydrate?
- Question 33: Which of the following foods is considered a simple carbohydrate?
- Question 34: In a hypoglycemic situation, which food would you consume first?
- Question 35: If you want to maintain stable glucose levels over a long period, which food would you consume?

As shown in Table 2, women with GDM who answered the different questions incorrectly—thus demonstrating erroneous knowledge regarding glycaemic control during pregnancy and the influence of diet on this process—exhibited poorer values in the evaluated parameters. Specifically, they had a higher pre-pregnancy body weight and poorer glucose tolerance, as reflected by significantly higher plasma glucose levels during the oral glucose tolerance test (OGTT) compared with women with GDM who answered the questions correctly. In addition, these women presented lower basal insulin and ferritin levels, as well as a less favourable lipid profile, characterised by higher total cholesterol and lower HDL-cholesterol concentrations than those observed in women who provided correct responses.

**Figure 2:**
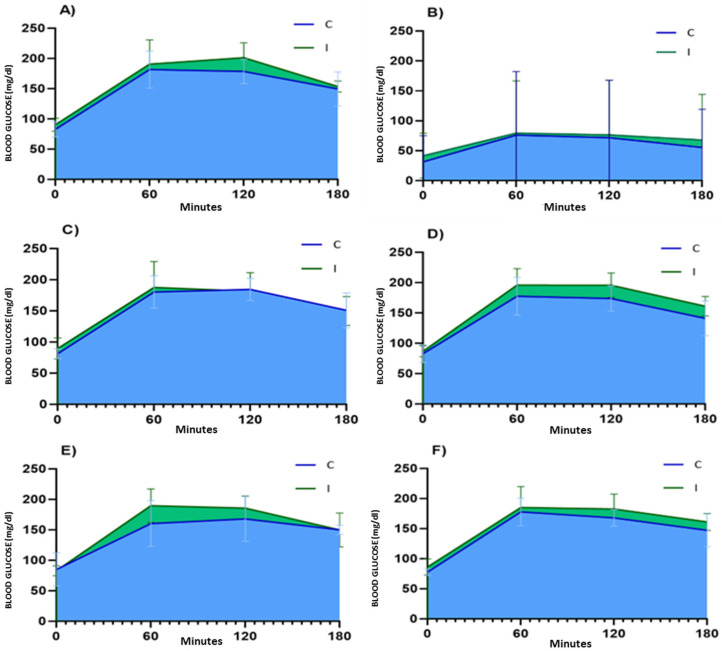
Analysis of the OGTT among women who answered correctly and incorrectly to the different questions assessing nutritional knowledge that may affect glycaemic control. The graphs show the mean ± standard deviation of plasma glucose concentrations for the entire cohort of women undergoing the OGTT, with measurements at baseline, 1, 2, and 3 h. Data are stratified according to whether participants answered correctly (C, blue) or incorrectly (I, green) to questions 22 (**A**), 23 (**B**), 24 (**C**), 25 (**D**), 30 (**E**), and 36 (**F**). A greater area under the curve can be observed in women who answered incorrectly.

The plasma glucose levels during the OGTT for women who answered the questions correctly and incorrectly are graphically depicted in Figure 2. To emphasise the differences between groups, the areas under the curve have been highlighted. As illustrated in the figure, women who answered incorrectly exhibited higher glucose concentrations throughout the OGTT compared with those who answered correctly, with a clear difference in the area under the curve across the questions analysed.

Secondly, differences in mean values are presented with the study cohort stratified according to the need for insulin or dietary treatment and according to whether participants answered the questions correctly or incorrectly (Table 3).

The table presents the mean values of the variables analysed across the different study groups. The *p* values also indicate the statistical differences between women who answered correctly and incorrectly to the different questions within each treatment category. Similarly, the results show that women who answered incorrectly—and therefore had poorer knowledge—exhibited higher plasma glucose levels, total cholesterol concentrations, and maternal pre-pregnancy weight, together with lower basal insulin, ferritin, and HDL-cholesterol levels.

Women who required insulin therapy received dual diabetes education. First, they were educated at the diabetes and pregnancy clinic at the time of GDM diagnosis, and second, they received additional education at the diabetes unit, where nutritional counselling aimed at achieving adequate glycaemic control was provided.

The following results show that women who answered incorrectly and required insulin treatment—having started from poorer baseline parameter values—achieved favourable perinatal outcomes. These findings suggest an association between the educational approach described for this group and favourable pregnancy outcomes. These data are presented in Table 4 (the overall cohort stratified by correct and incorrect responses) and in Table 5 (further stratified by insulin or dietary treatment).

**Figure 3:**
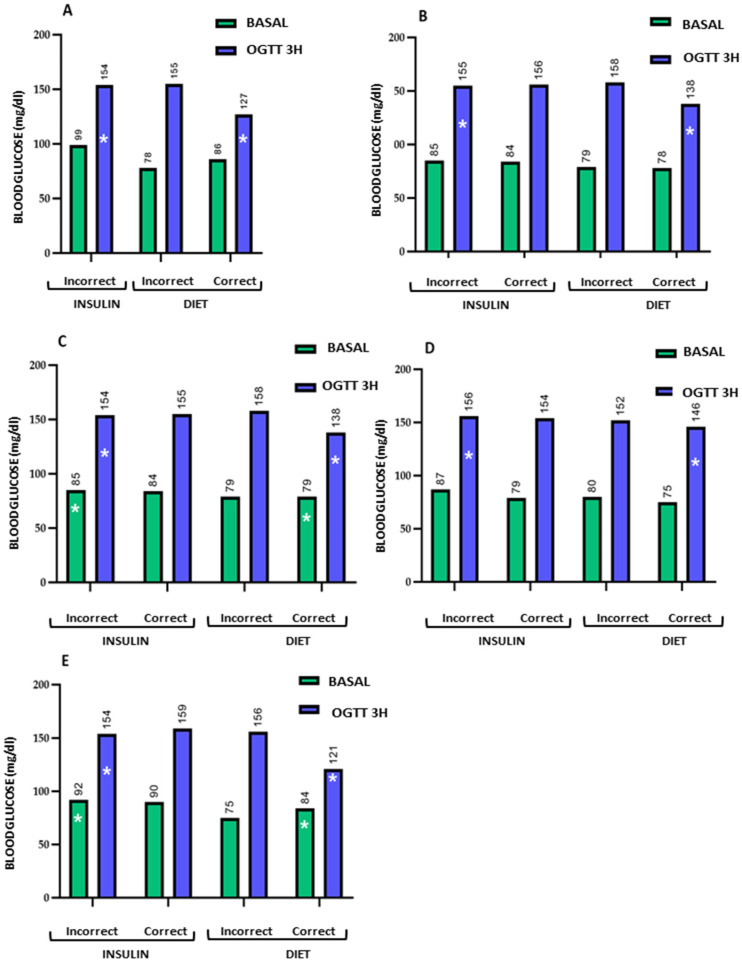
Graphical representation of fasting glucose and the 3 h OGTT in pregnant women receiving insulin or dietary treatment. The figure illustrates the differences in mean plasma glucose levels between women who answered correctly (C) and incorrectly (I) to the evaluated questions 23 (**A**), 31 (**B**), 33 (**C**), 34 (**D**), and 35 (**E**). Women who answered incorrectly and were treated with insulin tended to have higher glucose levels than those who answered correctly and received dietary treatment. To facilitate visualization, these differences are indicated with an asterisk (*). The numbers above the bars represent the mean glucose level in each study group. Quantitative data are shown as mean values; data variability (mean ± standard deviation) is reported in the corresponding tables.

**Figure 4:**
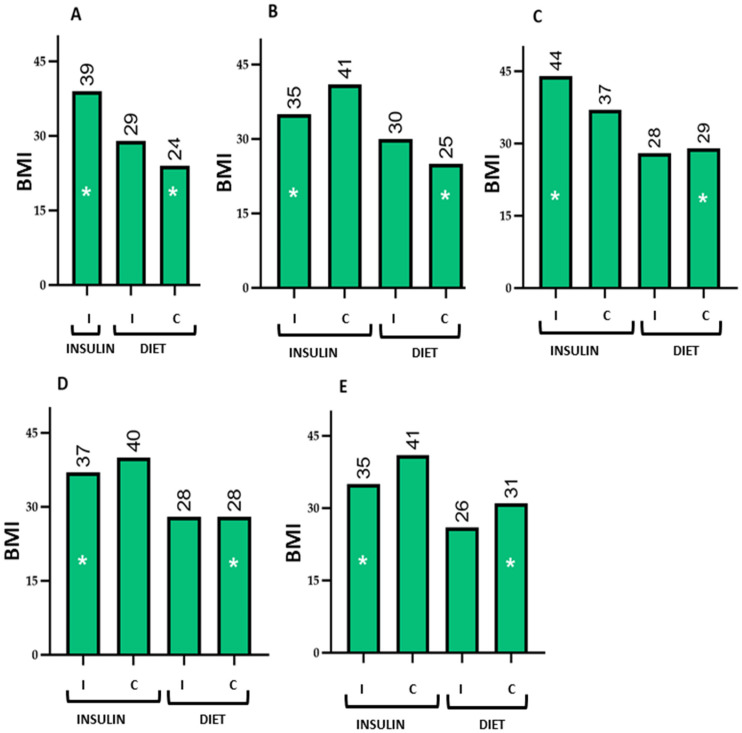
Graphical representation of BMI in pregnant women receiving insulin or dietary treatment, stratified by correct (C) or incorrect (I) responses. The figure shows the differences in mean BMI between the groups for the evaluated questions 23 (**A**), 31 (**B**), 33 (**C**), 34 (**D**), and 35 (**E**). Women who answered incorrectly and were treated with insulin tended to have higher BMI values than those who answered correctly and received dietary treatment. To facilitate visualization, these differences are indicated with an asterisk (*). The numbers above the bars represent the mean BMI in each study group. Quantitative data are shown as mean values; data variability (mean ± standard deviation) is reported in the corresponding tables.

**Figure 5:**
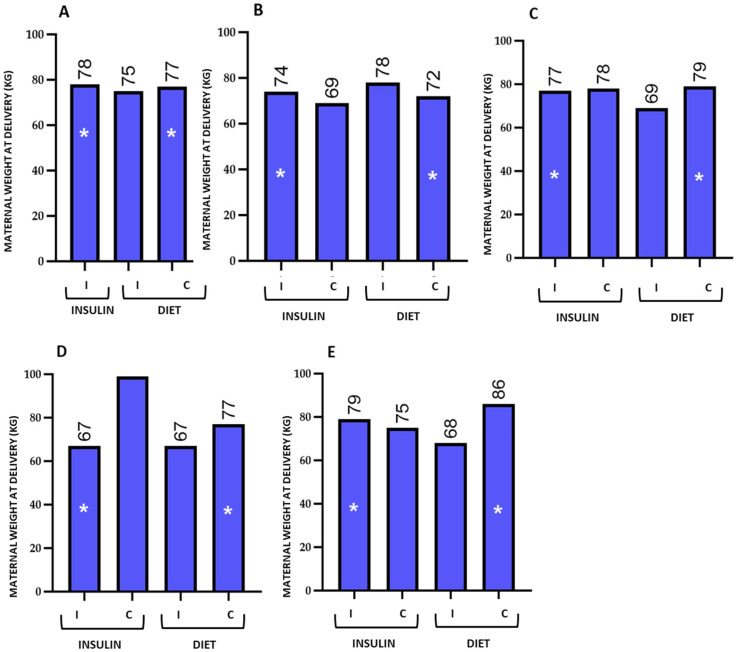
Graphical representation of maternal final weight in pregnant women receiving insulin or dietary treatment, stratified by correct (C) or incorrect (I) responses. The figure shows the differences in mean maternal weight between the groups for the evaluated questions 23 (**A**), 31 (**B**), 33 (**C**), 34 (**D**), and 35 (**E**). Women who answered incorrectly and were treated with insulin showed favourable outcomes at the end of pregnancy compared with those who received dietary treatment and answered correctly. To facilitate visualization, these differences are indicated with an asterisk (*). The numbers above the bars represent the mean maternal weight at the end of pregnancy in each study group. Quantitative data are shown as mean values; data variability (mean ± standard deviation) is reported in the corresponding tables.

**Figure 6:**
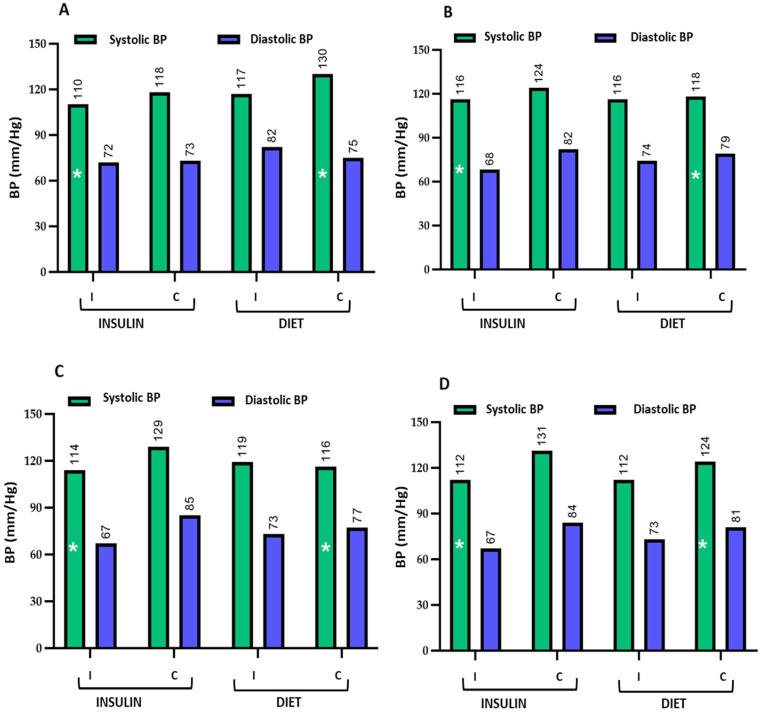
Graphical representation of maternal systolic and diastolic blood pressure (BP) in pregnant women receiving insulin or dietary treatment, stratified by correct (C) or incorrect (I) responses. The figure illustrates the differences in mean BP between groups for the evaluated questions 31 (**A**), 33 (**B**), 34 (**C**), and 35 (**D**). Women who answered incorrectly and were treated with insulin showed favourable outcomes at the end of pregnancy compared with those who received dietary treatment and answered correctly. To facilitate visualization, these differences are indicated with an asterisk (*). The numbers above the bars represent the mean systolic and diastolic BP values in each study group. Quantitative data are shown as mean values; data variability (mean ± standard deviation) is reported in the corresponding tables.

The table presents the mean values and standard deviations of the evaluated parameters among women receiving insulin or dietary treatment, stratified by whether they answered correctly or incorrectly. Figure 3, Figure 4, Figure 5 and Figure 6 graphically depict these values.

The comparison of means shows that women receiving insulin therapy who answered incorrectly had poorer baseline values, indicating lower nutritional knowledge, which could influence their lifestyle. Notably, these women still achieved favourable perinatal outcomes, even surpassing those of women who answered correctly and received dietary treatment. This suggests an association between the additional education provided to women receiving insulin therapy who answered incorrectly and the achievement of favourable pregnancy outcomes.

#### 3.5.2. Questions on Insulin Treatment

As previously mentioned, the group of women who required insulin therapy received dual diabetes education. First, they were educated at the diabetes and pregnancy clinic at the time of GDM diagnosis, and second, they received additional instruction at the diabetes unit, where nutritional education was provided to promote adequate glycaemic control.

This set of questions was answered exclusively by women receiving insulin therapy. It can be observed that these women had the poorest baseline parameter values, as expected given their diabetic status. However, they responded well to the questionnaire, indicating that the additional education they received was effectively internalized. Consistent with this, despite starting from worse baseline parameters, these women achieved favourable perinatal outcomes. This finding suggests that the education was internalized and was associated with favourable pregnancy outcomes.

#### 3.5.3. Questions on Physical Activity

Regarding the questions on physical activity, the results were consistent with those observed in the previous sections of the questionnaire. In this section, it is evident that women who answered incorrectly about exercise practice had poorer baseline parameter values. Perinatal outcomes, however, were favourable.

A binary logistic regression analysis was performed using the need for insulin as the dependent variable and selected demographic variables (specifically, level of education, occupation, parity, and BMI) as independent variables. No statistically significant predictive model for insulin requirement was identified.

Thus, the findings indicate that women who answered correctly had better initial values. Additionally, women who answered incorrectly and required insulin treatment—starting from poorer baseline parameters—still achieved favourable perinatal outcomes, suggesting an association between the dual education received by this group and favourable pregnancy outcomes.

## 4. Discussion

The present study demonstrates that nutritional knowledge and dietary management of glycaemic control in women with gestational diabetes mellitus (GDM) are significantly associated with maternal metabolic profile and key glucose tolerance parameters. Pregnant women with greater knowledge regarding nutrition, glycaemic index and load, recognition of hypoglycaemia, and carbohydrate management exhibited better fasting and post-load glucose values, lower pre-pregnancy weight, and a more favourable metabolic profile. These findings reinforce the hypothesis that nutritional literacy plays a crucial role in metabolic control of GDM, even prior to the initiation of pharmacological treatments.

The importance of glycaemic control during pregnancy has been widely established, with the Hyperglycemia and Adverse Pregnancy Outcome (HAPO) study demonstrating a continuous relationship between maternal glucose levels and the risk of adverse perinatal outcomes, even below traditional diagnostic thresholds [5]. International organizations such as the ADA, FIGO, and WHO emphasize the need for early diagnosis and appropriate management of GDM to minimize maternal and fetal complications [5,18,19]. Available evidence indicates that both prevention and optimization of GDM management should focus on modifying maternal risk factors, including excess pregestational weight, diet, and physical activity [20,21].

A key finding of this study is that women with lower nutritional knowledge exhibited poorer baseline values, including higher pre-pregnancy weight, increased BMI, and impaired glucose tolerance during the oral glucose challenge. These results align with evidence linking pregestational overweight and insulin resistance to increased risk of GDM and the need for pharmacological treatment [2,5,7]. Moreover, an unfavorable lipid profile—characterized by higher total cholesterol and lower HDL-cholesterol levels—suggests broader metabolic disturbances, likely influenced by inadequate dietary habits and lower physical activity prior to pregnancy. This underscores the importance of addressing metabolic risk factors comprehensively even before gestation.

Several studies and meta-analyses have demonstrated that lifestyle-based interventions—particularly structured nutritional education and promotion of physical activity—improve glycaemic control and reduce the need for insulin therapy in GDM [15,22,23]. However, most do not specifically assess the level of nutritional knowledge among pregnant women or its direct association with objective biochemical and clinical parameters. Our study provides novel evidence showing that nutritional knowledge is associated with improved metabolic parameters and pregnancy outcomes, independently of pharmacological intervention. Furthermore, it highlights that education per se exerts a reinforcing effect on the adoption of healthy habits, reflected in measurable changes in glucose levels and lipid profile.

Despite starting from a less favourable metabolic profile, women requiring insulin achieved adequate perinatal outcomes. This reflects the positive impact of intensified diabetes and nutritional education following diagnosis. Structured education appears to compensate for initial metabolic disadvantages, enabling effective glycaemic control and obstetric outcomes that are comparable—or even superior—to those of women treated exclusively with diet [15,24,25]. These findings underscore the importance of early and continuous educational interventions as an essential complement to any pharmacological strategy, contributing not only to glycaemic control but also to the prevention of perinatal complications.

Regarding physical activity, women with lower knowledge about exercise during pregnancy exhibited poorer metabolic parameters. This aligns with evidence identifying physical activity as a key modulator of insulin sensitivity and glucose metabolism [17,21]. Promotion of physical activity, together with targeted nutritional education, constitutes a fundamental pillar for improving both metabolic and perinatal health. The integration of these two educational components in GDM management allows for addressing risk factors from multiple angles and optimizing both maternal and neonatal outcomes.

From a clinical perspective, our findings emphasize the need to incorporate personalized nutritional and diabetes education programs into standard GDM management, as recommended by the ADA, FIGO, IDF, and national SEGO guidelines [1,2,6,7]. Beyond pharmacological treatment, empowering pregnant women with specific knowledge represents a key tool to optimize metabolic control and improve maternal-fetal outcomes. This approach not only benefits the mother during pregnancy but may also have long-term effects on neonatal health, given the role of intrauterine metabolism in the future development of disease.

Among the limitations of this study, the overall sample size and, particularly, the number of women requiring insulin treatment were relatively limited. However, the study was adequately powered for the primary outcome, as confirmed by the a priori sample size calculation, and the observed associations were statistically significant and clinically coherent. The observational design does not allow for causal inference but provides valuable evidence in a real-world clinical context. Conducting the study at a single center may limit generalizability; however, these findings support the need for multicenter studies with larger samples and prospective designs to confirm the effects of nutritional and diabetes education on glycaemic control and maternal–fetal outcomes in GDM. Future research could also evaluate the impact of preconception educational interventions, given the potential influence of pregestational nutrition on fetal programming and GDM risk.

Overall, the results provide robust evidence on the importance of nutritional knowledge and diabetes education as central pillars in the non-pharmacological management of GDM, highlighting their role in prevention, treatment, and optimization of pregnancy outcomes. These findings reinforce the need to integrate structured education as an essential component of any clinical protocol, contributing to improved maternal and neonatal health and to the reduction of complications associated with GDM.

## 5. Conclusions

Dietary and lifestyle knowledge have a significant impact on metabolic control in women diagnosed with gestational diabetes mellitus (GDM). Pregnant women with lower nutritional knowledge exhibit higher body weight, body mass index (BMI), and glucose levels in GDM diagnostic tests compared with women with greater knowledge.

This study provides novel evidence on the effectiveness of dual nutritional and diabetes education provided to women with GDM undergoing insulin therapy. The results show that, despite starting from a less favourable metabolic profile, these women achieve adequate maternal-fetal perinatal outcomes, even surpassing those observed in women who did not receive intensive education. These findings clearly demonstrate that structured education not only promotes the adoption of healthy habits but also has a direct effect on improving clinical outcomes, serving as an essential complementary tool to pharmacological treatment.

The findings underscore the need to develop preventive programs targeting women at risk for GDM, such as those of advanced maternal age, with a family history of diabetes mellitus, or with previous pregnancies complicated by GDM. Such programs should promote a healthy lifestyle, regular physical activity, and acquisition of specific nutritional knowledge for weight management and glycaemic control.

In conclusion, this study demonstrates that nutritional and diabetes education, provided before and during pregnancy, is both effective and essential for optimizing metabolic control and maternal-fetal outcomes in women with GDM. Furthermore, it provides novel evidence highlighting the importance of systematically evaluating the education delivered in obstetric and endocrinology clinics—an aspect that has been insufficiently documented in the scientific literature to date.

## Figures and Tables

**Table 1:** Questionnaire items on glycemic control management.

Identification data. Demographic data Anthropometric data Medical history Questions on glycemic control

**Table 2:** Initial parameters showing statistically significant differences between women with GDM who answered correctly versus incorrectly on the questionnaire regarding knowledge of the influence of diet on glycemic control.

Question	Variable	Incorrect	Correct	*p* Value
		Mean ± SD	Mean ± SD	
Nº 20	Total daily glucose measurements	5.2 ± 1.2	6.0 ± 0.0	0.05
	1-h glucose—100 g OGTT (mg/dL)	179.1 ± 29.8	163.5 ± 49.4	0.03
	Basal insulin (U/mL)	7.8 ± 3.1	10.8 ± 3.1	0.03
Nº 22	Fasting glucose—100 g OGTT (mg/dL)	90.2 ± 10.8	82.8 ± 12.7	0.06
	2-h glucose—100 g OGTT (mg/dL)	201.1 ± 27.7	178.3 ± 20.3	0.03
Nº 24	Maternal pre-pregnancy weight (kg)	73.7 ± 17.4	62.0 ± 14.1	0.03
Nº 25	2 h glucose—100 g OGTT (mg/dL)	195.4 ± 20.9	173.6 ± 20.9	0.01
Nº 25 Nº 27	Total protein (g/dL)	6.3 ± 0.4	6.1 ± 0.5	0.08
	Ferritin (mg/L)	10.2 ± 6.4	17.1 ± 11.2	0.03
	2 h glucose—100 g OGTT (mg/dL)	160.8 ± 16.1	141 ± 28.6	0.02
	1 h glucose—100 g OGTT (mg/dL)	189.4 ± 27.6	160.2 ± 37.5	0.06
Nº 28	3 h glucose—100 g OGTT (mg/dL)	152.9 ± 21.9	72.0 ± 0.0	0.06
Nº 29	1 h glucose—100 g OGTT (mg/dL)	198.5 ± 29.6	180.1 ± 32.4	0.04
Nº 29	Maternal weight (kg)	88.8 ± 11.9	72.5 ± 16.2	0.05
Nº 31	Previous child birth weight (kg)	3.410 ± 0.548	3.390 ± 0.609	0.04
Nº 32	Total cholesterol (mg/dL)	253.4 ± 39.7	246.8 ± 47.3	0.03
Nº 33	Basal insulin (U/mL)	7.0 ± 2.2	9.0 ± 3.4	0.04
Nº 33	3 h glucose—100 g OGTT (mg/dL)	158.1 ± 22.4	140.4 ± 27.0	0.04
Nº 34	Total daily glucose measurements	4.7 ± 1.4	5.8 ± 0.6	0.01
Nº 35	HDL cholesterol (mg/dL)	70.7 ± 16.6	87.6 ± 59.9	0.03

**Table 3:** Baseline parameters showing statistically significant differences between women treated with insulin or diet who answered the questions of the questionnaire on knowledge of the influence of diet on glycaemic control correctly or incorrectly.

Question	Variable	Insulin		Diet		*p* Value
		Incorrect	Correct	Incorrect	Correct	
		Meen ± SD	Mean ± SD	Mean ± SD	Mean ± SD	
Nº 20	Total daily glucose measurements	6.0 ± 0.0	6.0 ± 0.0	5.0 ± 1.26	6.0 ± 0.0	0.05
	1 h glucose—OGTT (mg/dL)	171.6 ± 21.0	none	181.4 ± 32.2	163.5 ± 49.4	0.03
	Basal insulin (U/mL)	8 ± 4.0	11± 0.0	7.76 ± 2.9	10.7 ± 3.5	0.03
Nº 22	Fasting glucose—OGTT (mg/dL)	94.5 ± 7.7	77.2 ± 6.6	88.6 ± 12.2	83.7 ± 13.3	0.06
	2 h glucose—OGTT (mg/dL)	204 ± 12.7	176.5 ± 13.7	200 ± 29.5	178.6 ± 21.4	0.03
Nº 24	Maternal pre-pregnancy weight (kg)	88.0 ± 0.0	none	72.9 ± 17.6	61.7 ± 12.0	0.03
Nº 25	2 h glucose—OGTT (mg/dL)	191.3 ± 23.7	180 ± 14.5	196.5 ± 20.3	172.4 ± 22.0	0.01
	Ferritin (ng/dL)	14 ± 11.2	28 ± 18.5	9.2 ± 4.9	15 ± 8.5	0.08
	Total protein (g/dL)	6.45 ± 0.2	5.9 ± 0.2	6.1 ± 0.3	6.2 ± 0.4	0.03
	3 h glucose—OGTT (mg/dL)	156.6 ± 2.3	155 ± 5	161.9 ± 17.9	138.3 ± 30.5	0.02
Nº 27	1 h glucose—OGTT (mg/dL)	169.2 ± 23.6	176.5 ± 21.9	192.5 ± 27.2	149.3 ± 46.2	0.06
Nº 28	3 h glucose—OGTT (mg/dL)	155.8 ± 3.6	72	152.3 ± 24.3	none	0.06
Nº 31	Previous child birth weight (kg)	2.956 ± 0.5	none	3.501 ± 0.51	3.390 ± 0.6	0.04
Nº 32	Total cholesterol (mg/dL)	294 ± 40.9	235.6 ± 35.9	238.6 ± 28.3	248.3 ± 49.0	0.03
Nº 33	Basal insulin (U/mL)	7.3 ± 3.2	10.5 ± 4.9	6.9 ± 2.1	8.8 ± 3.3	0.04
Nº 33	3 h glucose—OGTT (mg/dL)	155.7 ± 4.3	156 ± 2.8	158.8 ± 25.3	138.0 ± 28.4	0.04
Nº 34	Total daily glucose measurements	6.0 ± 0	6.0 ± 0.0	4.4 ± 1.43	5.7 ± 0.6	0.01
Nº 35	HDL cholesterol (mg/dL)	65.4 ± 16.4	76.0 ± 0.0	72.8 ± 16.9	88.5 ± 62.4	0.03

**Table 4:** Comparison of mean values between baseline and final parameters in women who answered correctly and incorrectly to selected questions assessing nutritional knowledge.

Questions			Initial Parameters											Final Parameters								
			Maternal Weight	Maternal Pre-Pregnancy Weight	BMI	Fasting O’Sullivan Test	1 h O’Sullivan Test	OGTT (100 g)	1 h OGTT (100 g)	2 h OGTT (100 g)	3 h OGTT (100 g)	Ferritin	Area Under the Curve of OGTT	Maternal Weight at Delivery	Systolic BP	Diastolic BP	Gestational Age at Delivery	Neonate Weight	Neonate Length	1 min Apgar Score	5 min Apgar Score	Weight Percentile
Nº 22	Correct	Mean	74.2	65.66	28.9	77.8	161.4	82.8	181.48	178.3	149.4	14.8	28,556	76.1	117	76.2	37.8	3170.3	48.4	8.3	9.5	61.6
		SD	16.5	16.42	6.6	7.6	31.0	12.7	30.8	20.3	28.1	10.4	1820	16.6	14.5	12.6	2.2	645.7	4.76	1.4	0.6	29.9
	Incorrect	Mean	76.3	69.5	31.4	81.2	158	90.2	190.43	201.1	153.4	11.5	30,806	67.5	119.3	74.2	38.8	3427.8	49	8.7	9.6	65
		SD	17.9	10	7.3	11.9	27.7	10.8	40.14	24.7	9.1	7.8	2044	16.1	18.1	15.3	0.9	411.01	1.6	0.4	0.5	29.6
Nº 23	Correct	Mean	76.5	64.2	24.8	86.5	157.7	81.7	178.3	181.2	127.2	14.9	11,453	77.3	115.3	74.1	35.2	3363.3	48.8	7.6	9.5	57
		SD	21.6	18.67	3.4	6.4	30.5	6.5	23.4	27.1	32.9	10.4	6502	13.1	23.8	20.1	4.03	610.9	2.5	2.8	0.8	34.7
	Incorrect	Mean	78.7	68.21	30.8	89.3	172	84.4	180.1	181.6	155.5	9.7	12,615	75.7	119.5	77.5	38.5	3161.2	48.4	8.5	9.5	64.1
		SD	15.9	16.29	6.7	9	28.3	13.5	30.4	20.4	22.1	1.5	5933	17.1	12.3	10.7	1.2	642.7	4.8	0.8	0.5	27.9
Nº 24	Correct	Mean	70.2	62.0	28.7	79.1	149.7	81.0	180.0	184.2	150.8	13.4	28,813	75.8	115.5	74.9	37.6	3318.5	48.5	8.3	9.5	51.1
		SD	14.6	14.1	7.1	9.4	33.9	7.92	25.9	17.7	28.0	10.8	1596	16.6	15.4	13.6	2.5	665.9	5.5	1.6	0.6	31.4
	Incorrect	Mean	80.4	73.7	30.8	81	172.6	89.5	187.3	181.4	149.7	15	29,306	74.2	122.3	78.5	38.5	3041.3	48.4	8.4	9.4	73.2
		SD	17.8	17.4	6.6	8.1	19.9	16.8	41.7	29.9	23.2	9.1	2342	16.6	13.7	12	0.9	514.9	2.1	0.6	0.5	23.0
Nº 25	Correct	Mean	72.4	64.9	29.3	80.4	158.1	82.7	177.3	173.6	141	17.1	27,769	75.8	120	76.9	38.5	3416.3	49.8	8.3	9.4	66.1
		SD	16.3	15.5	5.5	10.6	26.01	14.9	30.9	20.9	28.6	11.2	1855	19.4	17.7	15.7	1.4	515.4	1.7	0.9	0.6	29.1
	Incorrect	Mean	78.4	69.4	30.5	77.7	162.6	86.7	195.8	195.4	160.8	10.2	30,903	73.8	115.5	75.5	37.6	2996.5	47.1	8.3	9.6	45.2
		SD	16.3	18.5	7.6	8.13	34.0	9.2	27.1	20.2	16.1	6.5	1540	12.1	10.9	8.0	2.8	661.7	5.8	1.8	0.6	22.4
Nº 30	Correct	Mean	74.8	66.6	28.4	80.6	141.5	84.8	160.2	167.8	149.8	14.2	28,286	87.5	126.3	84.3	38.3	3173.5	48.6	8.6	9.7	57.3
		SD	17.0	17.2	7.8	8.6	47.1	27.1	37.59	36.6	7.6	8.4	2385	12.0	18.8	14.2	1.21	521.8	2.1	1.1	0.4	30.6
	Incorrect	Mean	75.6	67.1	30.3	78.8	164.3	82.7	189,	185.5	149.7	14.1	29,446	72.0	116.2	74.6	38.0	3221.0	48.4	8.2	9.4	60.5
		SD	14.0	15.1	6.4	9.7	23.7	8.3	27.6	19.8	27.7	10.4	1603	16.1	13.8	12.0	2.3	648.8	4.8	1.4	0.6	29.1
Nº 36	Correct	Mean	69.8	64.6	27.9	77.2	156.5	77.6	177.6	167.7	147.0	11.1	27,469	79.8	123.3	79	38.0	3020.5	46.3	8.8	9.6	30.2
		SD	20.2	20.1	8.6	10.8	16.7	5.6	22.9	13.8	27.3	4.4	1222	18.3	22.8	19.3	1.2	721.2	7	0.3	0.5	11.5
	Incorrect	Mean	75.0	66.4	29.4	79.5	161	86.2	184.8	181.3	161	15.3	28,970	70.6	115.1	74.1	38.0	3327.7	49.3	8.1	9.4	66.6
		SD	14.71	14.9	5.9	9.3	31.6	13.5	34.7	24.8	13.9	11.2	1992	14.8	12.5	11.0	2.5	566.1	2.1	1.5	0.6	25.0

**Table 5:** Baseline and final parameters evaluated in women receiving insulin or dietary treatment who answered selected nutritional knowledge questions correctly or incorrectly.

Questions				Initial Parameters			Final Parameters								
				Fasting Glucose	3 h OGTT (100 g)	BMI	Maternal Weight at Delivery	Systolic BP	Diastolic BP	Gestational Age at Delivery	1 min Apgar Score	5 -min Apgar Score	Neonate Weight	Neonate Length	Weight Percentile
Nº 23	INSULIN	Incorrect	Mean	99.6	155.8	39.3	77	119.0	-	38.6	9.1	10.0	3269.0	-	73.0
			SD	3.0	3.6	4.49	25.5	13.9	-	1.0	0.4	0.0	673.9	2.8	-
	DIET	Incorrect	Mean	78.1	155.4	29.5	75.1	119.7	-	38.5	8.3	9.4	3138.8	48.3	63.4
			SD	14.0	25.3	6.1	15.0	12.2	-	1.3	0.8	0.5	648.8	5.2	29.0
		Correct	Mean	86.5	127.1	24.8	77.3	115.3	-	37.2	7.6	9.5	3363.3	48.8	57.0
			SD	2.0	32.9	3.4	13.1	23.8	-	4.0	2.8	0.8	610.9	2.5	34.7
Nº 31	INSULIN	Incorrect	Mean	82.0	155.6	35.8	74.9	119.3	72.6	37.5	9.3	10.0	2797.5	46.5	73.0
			SD	4.2	2.0	-	8.2	21.5	10.5	0.7	0.5	0.0	781.3	0.7	-
		Correct	Mean	90.0	156.0	41.1	69.4	118.6	73.3	39.2	9.0	10.0	3583.3	50.3	-
			SD	-	5.9	4.6	27.8	4.9	18.7	0.5	0.0	0.0	482.3	2.5	-
	DIET	Incorrect	Mean	80.4	156.6	30.1	78.3	117.8	82.2	38.1	8.36	9.55	3203.44	48.29	59.25
			SD	10.8	23.3	6.2	15.5	13.6	13.1	1.4	0.92	0.52	694.55	6.03	28.49
		Correct	Mean	77.5	134.8	25.5	72.1	120.4	75.3	37.8	8.11	9.37	3168.75	48.50	63.56
			SD	5.6	32.6	3.8	12.0	17.9	11.6	2.6	1.66	0.68	572.35	2.24	32.54
Nº 33	INSULIN	Incorrect	Mean	85.0	155.7	44.4	77.7	116.2	68.5	38.7	9.00	10.00	3525.00	49.50	-
			SD	-	4.3	-	31.7	17.0	15.0	1.2	0.00	0.00	410.73	2.68	-
		Correct	Mean	84.5	156.0	36.8	78.3	124.5	82.0	38.5	9.50	10.00	2245.00	46.00	73.00
			SD	7.7	2.8	1.4	15.2	3.5	1.4	0.7	0.71	0.00	-	-	-
	DIET	Incorrect	Mean	79.6	158.8	28.0	69.6	116.8	74.1	38.5	8.60	9.53	3210.62	49.20	59.30
			SD	10.6	25.3	7.1	15.0	15.5	14.4	1.0	0.51	0.52	470.15	2.30	29.75
		Correct	Mean	78.5	138.0	29.1	79.4	118.5	79.3	37.1	7.80	9.33	3182.00	47.78	59.00
			SD	5.1	28.4	5.0	13.1	16.3	11.8	3.1	1.90	0.72	754.88	6.06	30.91
Nº 34	INSULIN	Incorrect	Mean	87.5	156.2	37.9	67.2	114.0	67.0	38.5	9.00	10.00	3320.00	48.50	-
			SD	3.5	3.9	-	23.7	13.9	12.6	1.0	0.00	0.00	30.00	2.18	-
		Correct	Mean	79.0	154.0	40.1	99.3	129.0	85.0	39.0	9.50	10.00	3192.50	49.25	73.00
			SD	-	-	6.1	14.4	9.9	2.8	1.4	0.71	0.00	1339.97	4.60	-
	DIET	Incorrect	Mean	80.2	152.4	28.5	67.8	119.1	73.8	38.1	8.36	9.36	3329.09	48.95	71.13
			SD	11.1	21.5	6.7	13.9	17.4	16.4	1.4	0.92	0.67	436.43	2.22	30.34
		Correct	Mean	75.7	146.3	28.3	77.5	116.1	77.8	37.7	8.19	9.52	3150.55	48.24	53.00
			SD	7.5	32.4	5.8	14.7	14.8	10.9	2.6	1.60	0.60	687.68	5.45	28.28
Nº 35	INSULIN	Incorrect	Mean	92.0	154.2	35.8	79.9	112.7	67.5	38.0	9.25	10.00	2971.67	46.83	73.00
			SD	4.2	3.3	-	26.5	12.5	13.4	1.0	0.50	0.00	629.49	0.76	-
		Correct	Mean	90.0	159.0	41.1	75.9	131.5	84.0	39.3	9.00	10.00	3715.00	51.75	-
			SD	-	1.4	4.6	30.2	6.3	4.2	0.5	0.00	0.00	601.04	1.06	-
	DIET	Incorrect	Mean	75.6	156.2	26.6	68.7	112.9	73.2	37.7	7.95	9.29	3123.73	47.85	60.45
			SD	8.6	20.9	4.4	12.1	12.4	10.7	2.6	1.63	0.64	612.68	5.43	29.85
		Correct	Mean	84.8	121.5	31.5	86.2	124.6	81.9	38.4	8.82	9.82	3382.73	49.59	63.50
			SD	9.8	35.5	7.4	14.2	18.3	15.4	1.0	0.41	0.41	607.85	2.44	32.51

## Data Availability

The original contributions presented in the study are included in the article/Supplementary Materials, further inquiries can be directed to the corresponding author.

## Supplementary Materials

The following supporting information can be downloaded at: https://www.mdpi.com/article/10.3390/nu18030513/s1, File S1: Questionnaire for GDM.

## Appendix A

**Table A1:** Maternal anthropometric data, maternal biochemical parameters, and maternal and neonatal clinical outcomes on the day of delivery, evaluated in the entire cohort participating in the study.

**Baseline Maternal Anthropometry**	
Height (cm)	162.1 ± 5.6 (151–175)
Pre-pregnancy weight (Kg)	66.5 ± 16.4 (43–108)
BMI	29.5 ± 6.7 (20–44.4)
**Biochemical Parameters**	
Gestational age at delivery (weeks)	38 ± 2.1 (28–40)
Fasting glucose (mg/dL)	79.1 ± 9.3 (61–97)
O’Sullivan glucose (mg/dL)	160.1 ± 29.2 (94–201)
Fasting glucose—100 g OGTT (mg/dL)	84.3 ± 12.6 (68–140)
1 h glucose—100 g OGTT (mg/dL)	183.2 ± 32.3 (96–239)
2 h glucose—100 g OGTT (mg/dL)	182.8 ± 22.8 (111–242)
3 h glucose—100 g OGTT (mg/dL)	150.2 ± 25.4 (72–209)
Basal insulin (U/mL)	8.0 ± 3.1 (3–15)
HbA1c (%)	5.3 ± 0.5 (4.8–6.6)
Ferritin (ng/mL)	14.1 ± 9.9 (5–49)
Total protein (g/dL)	6.22 ± 0.4 (5.6–7.5)
Hemoglobin (g/dL)	11.9 ± 1.0 (8–15)
Total Cholesterol (mg/dL)	244 ± 48.2 (127–355)
HDL- Cholesterol (mg/dL)	77.8 ± 40.7 (44–281)
LDL- Cholesterol (mg/dL)	147 ± 42.6 (37–242)
Tryglicerides (mg/dL)	204.5 ± 67.05 (95–387)
Vitamin-D (nmol/L)	20 ± 4.9 (11–29)
**Maternal Outcomes**	
Maternal weight at delivery (kg)	74.6 ± 16.6 (49.9–109.5)
Systolic blood pressure (mmHg)	117.5 ± 15.1 (94–160)
Diastolic blood pressure (mmHg)	75.8 ± 13.0 (52–110)
Gestational age at delivery (weeks)	38.0 ± 2.1 (28–40)
**Neonatal Outcomes**	
Neonate weight (kg)	3210 ± 612.9 (992–4380)
Neonate length (cm)	48.5 ± 4.3 (26–54)
1 min Apgar score	8.3 ± 1.3 (2–10)
5 min Apgar score	9.5 ± 0.6 (8–10)
Weight percentile	62.1 ± 29.0 (18–99)

The mean and standard deviation are presented, along with the range, indicating the minimum and maximum values obtained for each parameter.
