# Supplementary material for: Impact of Nutritional and Diabetological Education on Glycemic Control and Obstetric and Perinatal Outcomes in Gestational Diabetes Mellitus
Source: Nutrients. 2026 Feb 2;18(3):513. doi: 10.3390/nu18030513 (PMC12899032; doi:10.3390/nu18030513)
Supplement: Supplementary file 1 [file nutrients-18-00513-s001.zip › nutrients-4100231-supplementary.pdf]

Hospital Universitario La Paz
SaludMadrid
Comunidad de Madrid

CEU Universidad San Pablo

OCTUBRE DE 2018

CUESTIONARIO PARA GESTATES

DIABETES GESTACIONAL

En cumplimiento de lo dispuesto en la ley 15/1999, de 13 de diciembre, de Protección de Datos Personales (LOPD), le informamos que los datos personales obtenidos para este estudio son confidenciales y serán tratados de conformidad con lo establecido en la legislación vigente. Sólo tendrán acceso a los mismos los investigadores del estudio y para los fines que se le han indicado anteriormente. En cualquier momento usted podrá ejercitar los derechos de acceso, rectificación, oposición y, en su caso, cancelación, comunicándolo por escrito con indicación de sus datos a IDiPAZ- Dr. José Luis Bartha, en el domicilio Paseo Castellana 261, 28046 de Madrid.

CUESTIONARIO PARA GESTANTES CON DIABETES GESTACIONAL

Mediante la siguiente encuesta se va a valorar los conocimientos básicos de la gestante acerca de la **diabetes gestacional** para el control y manejo de esta.

I. DATOS IDENTIFICATIVOS

Nombre y apellidos:

Domicilio:

Provincia:

Teléfono:

País de origen:

Tiempo de residencia en España:

Área de Salud:

II. DATOS DEMOGRÁFICOS (Rodee la opción correcta)

Estado civil: Soltera, Casada, Divorciada, Viuda

Trabajo: SI, NO.

Especifique cual y que puesto desempeña…………………………………………….......

Nivel de estudios: ……………………………………………………………………

III. DATOS ANTROPOMÉTRICOS

1. Número de semanas de gestación…………………………………………………………...
2. Número de hijos totales contando con éste……………………………………………….
3. Planifico su embarazo
   a) Si
   b) No
4. ¿Cuánto mide usted?...
5. ¿Cuál es su peso actual?..
6. ¿Cuál era su preso previo a la gestación?...
7. De acuerdo con su peso previo, puede decir que era:
   a) Delgada
   b) Peso adecuado
   c) Sobrepeso
   d) Obesa

IV. HISTORIA CLÍNICA

8. ¿Padece alguna enfermedad crónica?
   a) Si
   b) No

   Indique cual: ……………………………………………………………………......
9. ¿Tiene alergias? (Fármacos, alimentos, intolerancias alimentarias)

   Indique cuales………………………………………………………………………
10. ¿Tiene familiares directos con diabetes o hipertensión?
    a) Si
    b) No

    Indique quien: ……………………………………………………………………
11. ¿Ha tenido problemas de glucosa o presión arterial previos al embarazo?
    a) Si
    b) No
12. ¿En embarazos previos tuvo diabetes gestacional o problemas de intolerancia a la glucosa?
    a) Si
    b) No

En caso de haber padecido diabetes gestacional en embarazos previos:

13. ¿Pudo controlar sus glucemias mediante dieta y ejercicio físico?
    a) Si
    b) No
14. ¿Preciso tratamiento con insulina?
    a) Si
    b) No
15. En caso de tener conocimiento de ello, indique que tipo de insulina tuvo durante el tratamiento:
    a) Insulina de acción intermedia NPH
    b) Insulina de acción Lenta
    c) Insulina de acción Rápida
    d) Tratamiento combinado de Insulina de acción Lenta y Rápida
    e) Insulina tipo (Mezcla)
16. En caso de tener conocimiento de ello. Indique que nivel de hemoglobina glicosilada (HbA1c) tuvo durante la gestación o en su último control: ……………………………………………………………………………..
17. ¿Controlaba sus niveles de glucosa mediante la realización de glucemias pre y postprandiales?
    a) Si
    b) No

18. ¿Cuánto peso su anterior hijo al nacer?..
19. ¿Su anterior parto fue natural o cesarea?..

V. CUESTIONES PARA EL CONTROL GLUCÉMICO

Glucemia= Nivel de "azúcar" en sangre
Hidratos de carbono= Azúcar presente en los alimentos
Preprandial= Antes de las comidas
Postprandial= Después de las comidas

20. ¿Cuántas glucemias al día te realizas?
    a) Pre y postprandiales
    b) Antes de las principales comidas
    c) Pre y postprandiales y ante síntomas de hipoglucemia
    Totales al día………………………………………………………………………
21. ¿Cuáles es el valor/es de glucemia óptimos para el desarrollo fetal y salud materna?

a) 70-120 mg/dl

b) Niveles inferiores a 140 mg/dl

c) 95 mg/dl antes de las principales comidas

22. ¿Cuándo hablamos de hipoglucemia nos referíamos a?

a) Bajada de azúcar

b) Subida de azúcar

23. ¿Qué nivel es considerado como hipoglucemia?

a) < 70 mg/dl

b) < 60 mg/dl

c) < 65 mg/dl

24. ¿Cuáles de los siguientes son síntomas de hipoglucemia?

a) Sudoración, taquicardia, debilidad en miembros inferiores, hambre, cansancio, mareo

b) Boca seca, calor, sed extrema, náuseas, micción frecuente

25. ¿Qué es el índice glucémico de un alimento?

a) Velocidad a la que un alimento aumenta los niveles de glucosa en sangre con misma carga glucémica.

b) Cantidad de azucares o carbohidratos presentes en un alimento teniendo en cuenta el tamaño de la ración.

26. ¿Qué es la carga glucémica de un alimento?

a) Velocidad a la que un alimento aumenta los niveles de glucosa en sangre con misma carga glucémica.

b) Cantidad de azucares o carbohidratos presentes en un alimento teniendo en cuenta el tamaño de la ración.

27. ¿Cuántos gramos de hidratos de carbono son considerados una ración de estos?

a) 10 gr de Hidratos de carbono

b) 20 gr de Hidratos de carbono

c) 15 gr de Hidratos de carbono

28. ¿Cuál de los siguientes alimentos consideras que tiene un índice glucémico mayor?

a) Kiwi

b) Plátano

c) Manzana

29. ¿Cuál de los siguientes alimentos consideras que tiene una carga glucémica mayor?

a) Kiwi
b) Plátano
c) Manzana

30. De forma general, ¿Qué porcentaje de las calorías totales diarias de la gestante deben ser en forma de hidratos de carbono?
    a) 50-60%
    b) 30%
    c) 20%
31. ¿Ante una situación de hipoglucemia que alimentos ingeriría primero?
    a) Alimentos con mayor índice glucémico
    b) Alimentos con mayor carga glucémica
32. ¿Qué es un hidrato de carbono simple?
    a) Azucares de absorción rápida, que nos proporcionan aumento de la glucemia y energía de forma inmediata, pero de corta duración en el tiempo.
    b) Azucares de absorción lenta, mantienen los niveles de glucemia más estables y de larga duración.
33. ¿Cuál de los siguientes alimentos considera que es un hidrato de carbono simple?
    a) Miel
    b) Legumbres
    c) Patata
34. ¿Ante una situación de hipoglucemia que alimento consumiría primero?
    a) Miel
    b) Pan blanco
    c) Fruta
35. ¿Si desea mantener los niveles de glucemia estables durante un periodo largo, que alimento consumiría?
    a) Verdura
    b) Legumbres
    c) Zumos de frutas naturales
36. En cuanto al etiquetado nutricional; si nos indica que determinado alimento contiene 35 gramos de carbohidratos, ¿Cuántas raciones de hidratos de carbono contendrá?
    a) 2 raciones de hidratos de carbono
    b) 35 raciones de hidratos de carbono
    c) 3 raciones y media de hidratos de carbono

SOLO SI USTED ESTÁ PRECISANDO TRATAMIENTO CON INSULINA RESPONDA ESTE APARTADO.

37. En el caso de estar precisando tratamiento con insulina NPH; ¿Se trata de una insulina de acción?
    a) Acción ultrarrápida
    b) Acción intermedia
    c) Acción lenta
38. ¿En qué momentos se administra la insulina de acción rápida/ultrarrápida?
    a) Una vez cada 24h, para mantener estables los niveles de glucosa basales y entre las comidas.
    b) Antes de las comidas para metabolizar así los azucares de la ingesta.
    c) Antes de las comidas para metabolizar así los azucares de la ingesta y antes situaciones de hiperglucemia o glucemias por encima de los valores requeridos.
39. La insulina de acción lenta se encarga de mantener estables los niveles de glucemia;
    a) Postprandiales (después de las comidas)
    b) Basales (en ayunas al despertarnos)
    c) Basales y entre las comidas cuando no existen efectos de otros tipos de insulina.

40. ¿Ante una situación de hipoglucemia de forma preprandial disminuiría los niveles de?
    a) Insulina de acción rápida
    b) Insulina de acción lenta
41. ¿Rota el lugar de punción al administrarse la insulina?
    a) Si, cambio el lugar de punción
    b) No, siempre la administro en el mismo lugar
42. ¿Las insulinas deben mantenerse refrigeradas y evitar exposiciones de calor excesivas?
    a) Si, deben sacarse del frigorífico 10 min antes de su uso
    b) No, pueden mantenerse en temperaturas inferiores a 25ºC cuando esta en uso.
    c) Ambas son ciertas
43. ¿Considera importante para el control glucémico y la salud materno-fetal la práctica de ejercicio físico?

a) Si, el ejercicio físico aeróbico ayuda a disminuir los niveles de glucemia, evita la ganancia de peso excesiva, controla los niveles de presión arterial de la gestante…
b) No, el ejercicio físico no influye en las glucemias

44. ¿Qué actividad física realiza?
    a) 20-30 min de ejercicio aeróbico al día (pasear, nadar.)
    b) 30 min de ejercicio aeróbico 2/3 días a la semana
    c) De forma esporádica
45. ¿Considera que el ejercicio físico influye en la glucemia?
    a) En el momento en el que se está realizando la actividad física
    b) Horas después de la actividad física
    c) En el momento de su realización, horas después, hasta 48h post realización de este.
